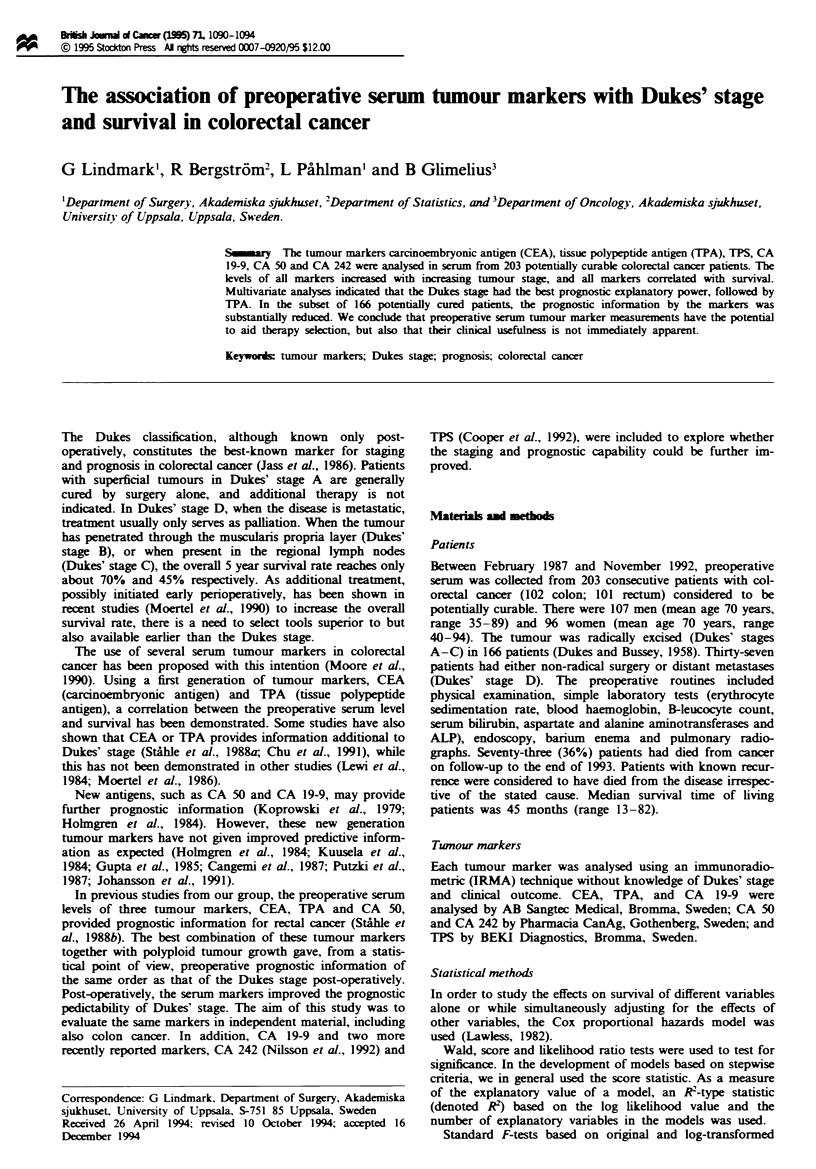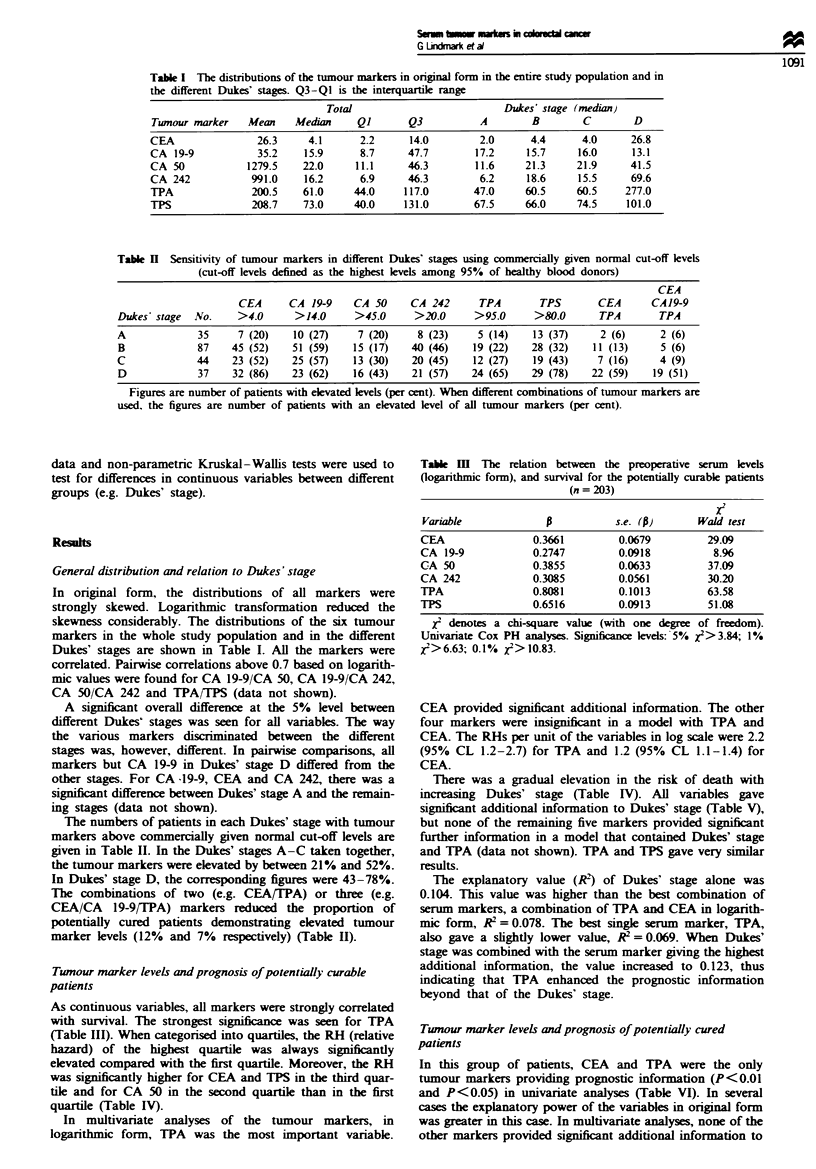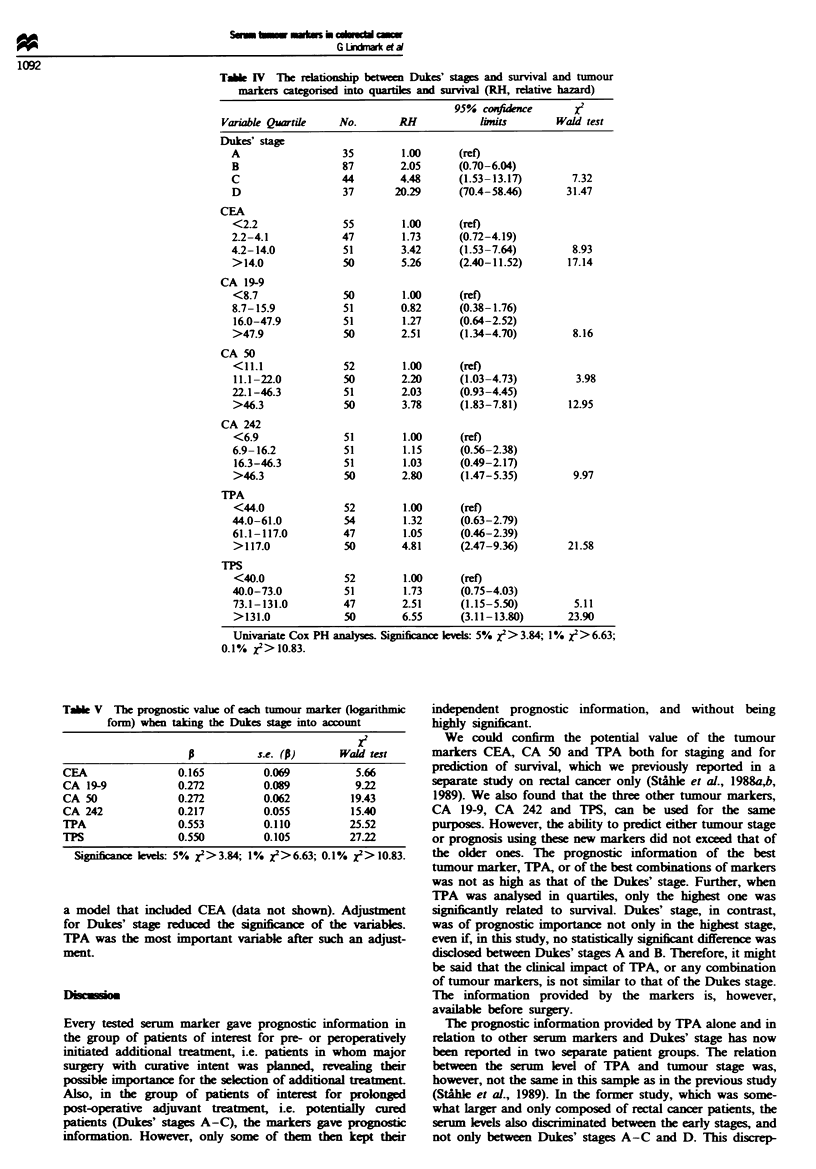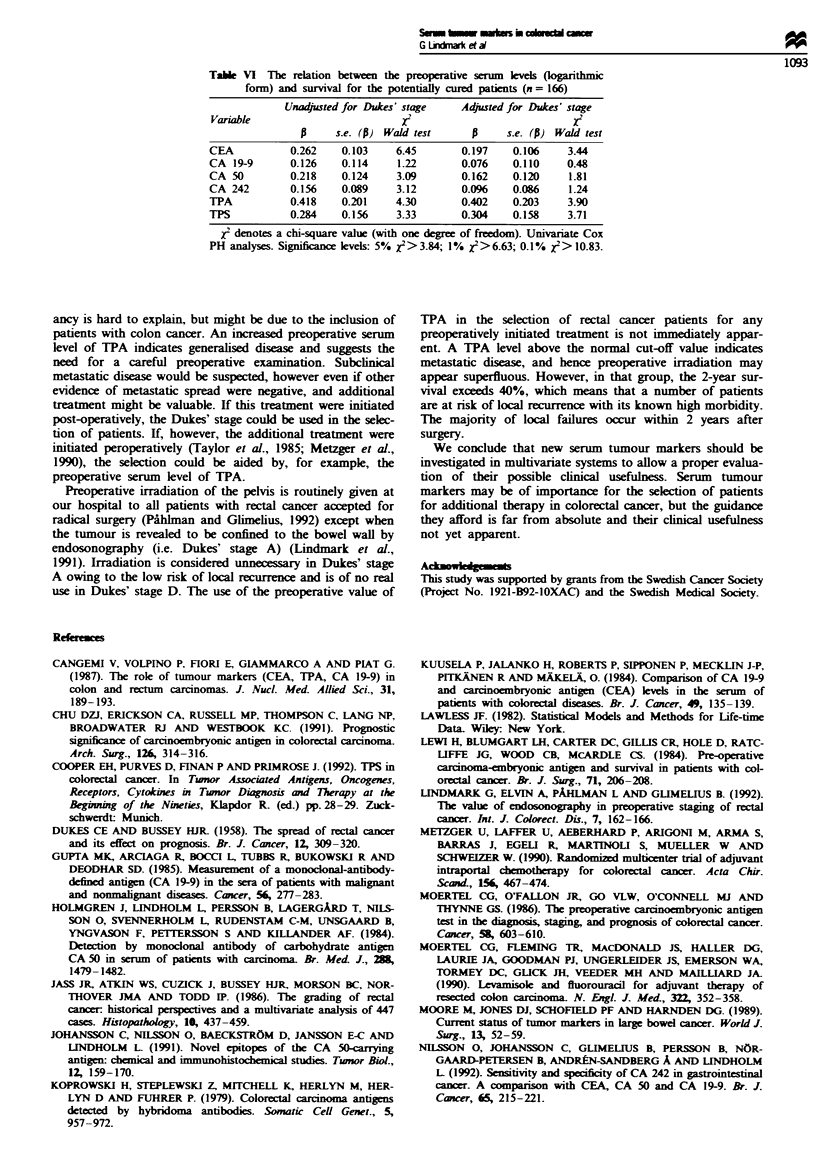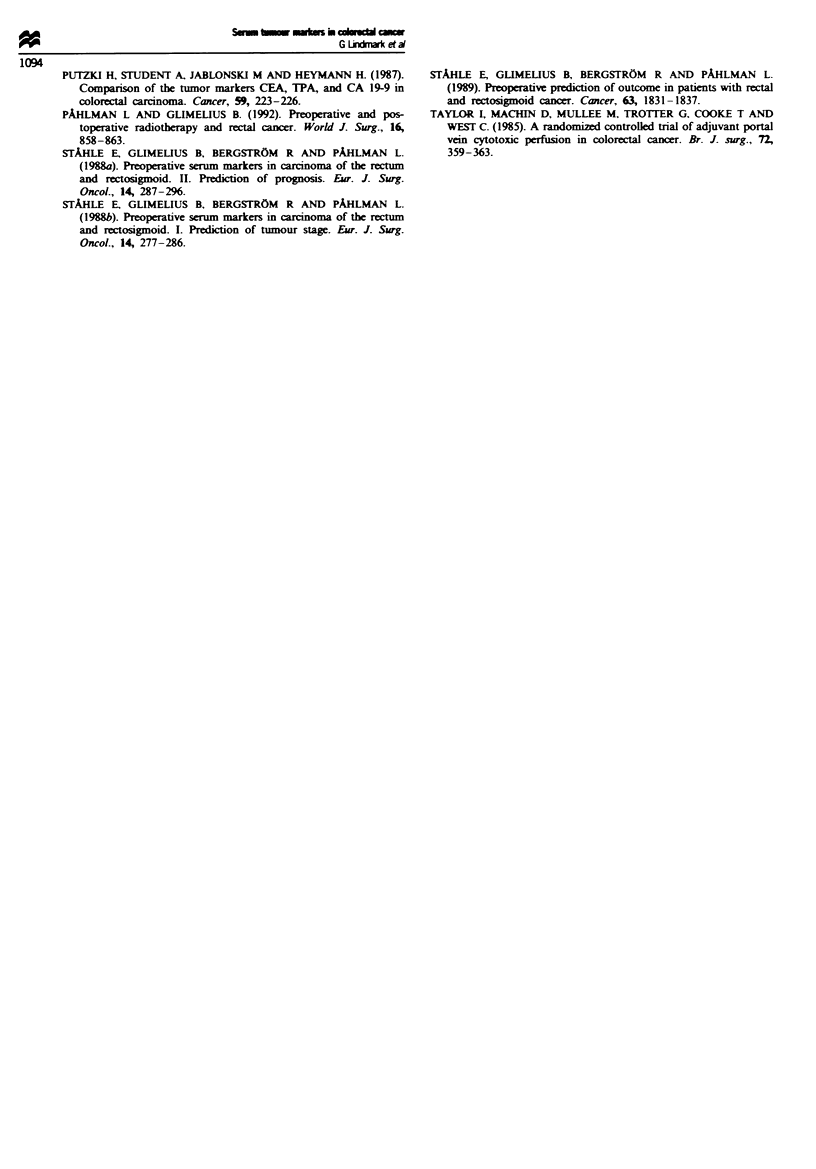# The association of preoperative serum tumour markers with Dukes' stage and survival in colorectal cancer.

**DOI:** 10.1038/bjc.1995.211

**Published:** 1995-05

**Authors:** G. Lindmark, R. Bergström, L. Påhlman, B. Glimelius

**Affiliations:** Department of Surgery, Akademiska sjukhuset, Uppsala, Sweden.

## Abstract

The tumour markers carcinoembryonic antigen (CEA), tissue polypeptide antigen (TPA), TPS, CA 19-9, CA 50 and CA 242 were analysed in serum from 203 potentially curable colorectal cancer patients. The levels of all markers increased with increasing tumour stage, and all markers correlated with survival. Multivariate analyses indicated that the Dukes stage had the best prognostic explanatory power, followed by TPA. In the subset of 166 potentially cured patients, the prognostic information by the markers was substantially reduced. We conclude that preoperative serum tumour marker measurements have the potential to aid therapy selection, but also that their clinical usefulness is not immediately apparent.


© 1995 Stockton Press All rights reserved 0007–0920/95 $12.00

### The association of preoperative serum tumour markers with Dukes' stage and survival in colorectal cancer

G Lindmark[1], R Bergström[2], L Påhlman[1] and B Glimelius[3]

[1]*Department of Surgery, Akademiska sjukhuset,* [2]*Department of Statistics, and* [3]*Department of Oncology, Akademiska sjukhuset, University of Uppsala, Uppsala, Sweden.*

**Summary** The tumour markers carcinoembryonic antigen (CEA), tissue polypeptide antigen (TPA), TPS, CA 19-9, CA 50 and CA 242 were analysed in serum from 203 potentially curable colorectal cancer patients. The levels of all markers increased with increasing tumour stage, and all markers correlated with survival. Multivariate analyses indicated that the Dukes stage had the best prognostic explanatory power, followed by TPA. In the subset of 166 potentially cured patients, the prognostic information by the markers was substantially reduced. We conclude that preoperative serum tumour marker measurements have the potential to aid therapy selection, but also that their clinical usefulness is not immediately apparent.

**Keywords:** tumour markers; Dukes stage; prognosis; colorectal cancer

The Dukes classification, although known only post-operatively, constitutes the best-known marker for staging and prognosis in colorectal cancer (Jass *et al.*, 1986). Patients with superficial tumours in Dukes' stage A are generally cured by surgery alone, and additional therapy is not indicated. In Dukes' stage D, when the disease is metastatic, treatment usually only serves as palliation. When the tumour has penetrated through the muscularis propria layer (Dukes' stage B), or when present in the regional lymph nodes (Dukes' stage C), the overall 5 year survival rate reaches only about 70% and 45% respectively. As additional treatment, possibly initiated early perioperatively, has been shown in recent studies (Moertel *et al.*, 1990) to increase the overall survival rate, there is a need to select tools superior to but also available earlier than the Dukes stage.

The use of several serum tumour markers in colorectal cancer has been proposed with this intention (Moore *et al.*, 1990). Using a first generation of tumour markers, CEA (carcinoembryonic antigen) and TPA (tissue polypeptide antigen), a correlation between the preoperative serum level and survival has been demonstrated. Some studies have also shown that CEA or TPA provides information additional to Dukes' stage (Ståhle *et al.*, 1988*a*; Chu *et al.*, 1991), while this has not been demonstrated in other studies (Lewi *et al.*, 1984; Moertel *et al.*, 1986).

New antigens, such as CA 50 and CA 19-9, may provide further prognostic information (Koprowski *et al.*, 1979; Holmgren *et al.*, 1984). However, these new generation tumour markers have not given improved predictive information as expected (Holmgren *et al.*, 1984; Kuusela *et al.*, 1984; Gupta *et al.*, 1985; Cangemi *et al.*, 1987; Putzki *et al.*, 1987; Johansson *et al.*, 1991).

In previous studies from our group, the preoperative serum levels of three tumour markers, CEA, TPA and CA 50, provided prognostic information for rectal cancer (Ståhle *et al.*, 1988*b*). The best combination of these tumour markers together with polyploid tumour growth gave, from a statistical point of view, preoperative prognostic information of the same order as that of the Dukes stage post-operatively. Post-operatively, the serum markers improved the prognostic pedictability of Dukes' stage. The aim of this study was to evaluate the same markers in independent material, including also colon cancer. In addition, CA 19-9 and two more recently reported markers, CA 242 (Nilsson *et al.*, 1992) and TPS (Cooper *et al.*, 1992), were included to explore whether the staging and prognostic capability could be further improved.

Correspondence: G Lindmark, Department of Surgery, Akademiska sjukhuset, University of Uppsala, S-751 85 Uppsala, Sweden
Received 26 April 1994; revised 10 October 1994; accepted 16 December 1994

### Materials and methods

### Patients

Between February 1987 and November 1992, preoperative serum was collected from 203 consecutive patients with colorectal cancer (102 colon; 101 rectum) considered to be potentially curable. There were 107 men (mean age 70 years, range 35–89) and 96 women (mean age 70 years, range 40–94). The tumour was radically excised (Dukes' stages A–C) in 166 patients (Dukes and Bussey, 1958). Thirty-seven patients had either non-radical surgery or distant metastases (Dukes' stage D). The preoperative routines included physical examination, simple laboratory tests (erythrocyte sedimentation rate, blood haemoglobin, B-leucocyte count, serum bilirubin, aspartate and alanine aminotransferases and ALP), endoscopy, barium enema and pulmonary radiographs. Seventy-three (36%) patients had died from cancer on follow-up to the end of 1993. Patients with known recurrence were considered to have died from the disease irrespective of the stated cause. Median survival time of living patients was 45 months (range 13–82).

### Tumour markers

Each tumour marker was analysed using an immunoradiometric (IRMA) technique without knowledge of Dukes' stage and clinical outcome. CEA, TPA, and CA 19-9 were analysed by AB Sangtec Medical, Bromma, Sweden; CA 50 and CA 242 by Pharmacia CanAg, Gothenberg, Sweden; and TPS by BEKI Diagnostics, Bromma, Sweden.

### Statistical methods

In order to study the effects on survival of different variables alone or while simultaneously adjusting for the effects of other variables, the Cox proportional hazards model was used (Lawless, 1982).

Wald, score and likelihood ratio tests were used to test for significance. In the development of models based on stepwise criteria, we in general used the score statistic. As a measure of the explanatory value of a model, an $R^2$-type statistic (denoted $\bar{R}^2$) based on the log likelihood value and the number of explanatory variables in the models was used.

Standard *F*-tests based on original and log-transformed

Table I The distributions of the tumour markers in original form in the entire study population and in the different Dukes' stages. Q3–Q1 is the interquartile range

| | Total | | | | Dukes' stage (median) | | | |
|---|---|---|---|---|---|---|---|---|
| Tumour marker | Mean | Median | Q1 | Q3 | A | B | C | D |
| CEA | 26.3 | 4.1 | 2.2 | 14.0 | 2.0 | 4.4 | 4.0 | 26.8 |
| CA 19-9 | 35.2 | 15.9 | 8.7 | 47.7 | 17.2 | 15.7 | 16.0 | 13.1 |
| CA 50 | 1279.5 | 22.0 | 11.1 | 46.3 | 11.6 | 21.3 | 21.9 | 41.5 |
| CA 242 | 991.0 | 16.2 | 6.9 | 46.3 | 6.2 | 18.6 | 15.5 | 69.6 |
| TPA | 200.5 | 61.0 | 44.0 | 117.0 | 47.0 | 60.5 | 60.5 | 277.0 |
| TPS | 208.7 | 73.0 | 40.0 | 131.0 | 67.5 | 66.0 | 74.5 | 101.0 |

Table II Sensitivity of tumour markers in different Dukes' stages using commercially given normal cut-off levels (cut-off levels defined as the highest levels among 95% of healthy blood donors)

| Dukes' stage | No. | CEA >4.0 | CA 19-9 >14.0 | CA 50 >45.0 | CA 242 >20.0 | TPA >95.0 | TPS >80.0 | CEA TPA | CEA CA19-9 TPA |
|---|---|---|---|---|---|---|---|---|---|
| A | 35 | 7 (20) | 10 (27) | 7 (20) | 8 (23) | 5 (14) | 13 (37) | 2 (6) | 2 (6) |
| B | 87 | 45 (52) | 51 (59) | 15 (17) | 40 (46) | 19 (22) | 28 (32) | 11 (13) | 5 (6) |
| C | 44 | 23 (52) | 25 (57) | 13 (30) | 20 (45) | 12 (27) | 19 (43) | 7 (16) | 4 (9) |
| D | 37 | 32 (86) | 23 (62) | 16 (43) | 21 (57) | 24 (65) | 29 (78) | 22 (59) | 19 (51) |

Figures are number of patients with elevated levels (per cent). When different combinations of tumour markers are used, the figures are number of patients with an elevated level of all tumour markers (per cent).

data and non-parametric Kruskal–Wallis tests were used to test for differences in continuous variables between different groups (e.g. Dukes' stage).

### Results

### *General distribution and relation to Dukes' stage*

In original form, the distributions of all markers were strongly skewed. Logarithmic transformation reduced the skewness considerably. The distributions of the six tumour markers in the whole study population and in the different Dukes' stages are shown in Table I. All the markers were correlated. Pairwise correlations above 0.7 based on logarithmic values were found for CA 19-9/CA 50, CA 19-9/CA 242, CA 50/CA 242 and TPA/TPS (data not shown).

A significant overall difference at the 5% level between different Dukes' stages was seen for all variables. The way the various markers discriminated between the different stages was, however, different. In pairwise comparisons, all markers but CA 19-9 in Dukes' stage D differed from the other stages. For CA 19-9, CEA and CA 242, there was a significant difference between Dukes' stage A and the remaining stages (data not shown).

The numbers of patients in each Dukes' stage with tumour markers above commercially given normal cut-off levels are given in Table II. In the Dukes' stages A–C taken together, the tumour markers were elevated by between 21% and 52%. In Dukes' stage D, the corresponding figures were 43–78%. The combinations of two (e.g. CEA/TPA) or three (e.g. CEA/CA 19-9/TPA) markers reduced the proportion of potentially cured patients demonstrating elevated tumour marker levels (12% and 7% respectively) (Table II).

### *Tumour marker levels and prognosis of potentially curable patients*

As continuous variables, all markers were strongly correlated with survival. The strongest significance was seen for TPA (Table III). When categorised into quartiles, the RH (relative hazard) of the highest quartile was always significantly elevated compared with the first quartile. Moreover, the RH was significantly higher for CEA and TPS in the third quartile and for CA 50 in the second quartile than in the first quartile (Table IV).

In multivariate analyses of the tumour markers, in logarithmic form, TPA was the most important variable.

Table III The relation between the preoperative serum levels (logarithmic form), and survival for the potentially curable patients ($n = 203$)

| Variable | $\beta$ | s.e. ($\beta$) | $\chi^2$ Wald test |
|---|---|---|---|
| CEA | 0.3661 | 0.0679 | 29.09 |
| CA 19-9 | 0.2747 | 0.0918 | 8.96 |
| CA 50 | 0.3855 | 0.0633 | 37.09 |
| CA 242 | 0.3085 | 0.0561 | 30.20 |
| TPA | 0.8081 | 0.1013 | 63.58 |
| TPS | 0.6516 | 0.0913 | 51.08 |

$\chi^2$ denotes a chi-square value (with one degree of freedom). Univariate Cox PH analyses. Significance levels: 5% $\chi^2 > 3.84$; 1% $\chi^2 > 6.63$; 0.1% $\chi^2 > 10.83$.

CEA provided significant additional information. The other four markers were insignificant in a model with TPA and CEA. The RHs per unit of the variables in log scale were 2.2 (95% CL 1.2–2.7) for TPA and 1.2 (95% CL 1.1–1.4) for CEA.

There was a gradual elevation in the risk of death with increasing Dukes' stage (Table IV). All variables gave significant additional information to Dukes' stage (Table V), but none of the remaining five markers provided significant further information in a model that contained Dukes' stage and TPA (data not shown). TPA and TPS gave very similar results.

The explanatory value ($R^2$) of Dukes' stage alone was 0.104. This value was higher than the best combination of serum markers, a combination of TPA and CEA in logarithmic form, $R^2 = 0.078$. The best single serum marker, TPA, also gave a slightly lower value, $R^2 = 0.069$. When Dukes' stage was combined with the serum marker giving the highest additional information, the value increased to 0.123, thus indicating that TPA enhanced the prognostic information beyond that of the Dukes' stage.

### *Tumour marker levels and prognosis of potentially cured patients*

In this group of patients, CEA and TPA were the only tumour markers providing prognostic information ($P < 0.01$ and $P < 0.05$) in univariate analyses (Table VI). In several cases the explanatory power of the variables in original form was greater in this case. In multivariate analyses, none of the other markers provided significant additional information to

Table IV The relationship between Dukes' stages and survival and tumour markers categorised into quartiles and survival (RH, relative hazard)

| Variable Quartile | No. | RH | 95% confidence limits | $\chi^2$ Wald test |
|---|---|---|---|---|
| Dukes' stage | | | | |
| A | 35 | 1.00 | (ref) | |
| B | 87 | 2.05 | (0.70-6.04) | |
| C | 44 | 4.48 | (1.53-13.17) | 7.32 |
| D | 37 | 20.29 | (70.4-58.46) | 31.47 |
| CEA | | | | |
| <2.2 | 55 | 1.00 | (ref) | |
| 2.2-4.1 | 47 | 1.73 | (0.72-4.19) | |
| 4.2-14.0 | 51 | 3.42 | (1.53-7.64) | 8.93 |
| >14.0 | 50 | 5.26 | (2.40-11.52) | 17.14 |
| CA 19-9 | | | | |
| <8.7 | 50 | 1.00 | (ref) | |
| 8.7-15.9 | 51 | 0.82 | (0.38-1.76) | |
| 16.0-47.9 | 51 | 1.27 | (0.64-2.52) | |
| >47.9 | 50 | 2.51 | (1.34-4.70) | 8.16 |
| CA 50 | | | | |
| <11.1 | 52 | 1.00 | (ref) | |
| 11.1-22.0 | 50 | 2.20 | (1.03-4.73) | 3.98 |
| 22.1-46.3 | 51 | 2.03 | (0.93-4.45) | |
| >46.3 | 50 | 3.78 | (1.83-7.81) | 12.95 |
| CA 242 | | | | |
| <6.9 | 51 | 1.00 | (ref) | |
| 6.9-16.2 | 51 | 1.15 | (0.56-2.38) | |
| 16.3-46.3 | 51 | 1.03 | (0.49-2.17) | |
| >46.3 | 50 | 2.80 | (1.47-5.35) | 9.97 |
| TPA | | | | |
| <44.0 | 52 | 1.00 | (ref) | |
| 44.0-61.0 | 54 | 1.32 | (0.63-2.79) | |
| 61.1-117.0 | 47 | 1.05 | (0.46-2.39) | |
| >117.0 | 50 | 4.81 | (2.47-9.36) | 21.58 |
| TPS | | | | |
| <40.0 | 52 | 1.00 | (ref) | |
| 40.0-73.0 | 51 | 1.73 | (0.75-4.03) | |
| 73.1-131.0 | 47 | 2.51 | (1.15-5.50) | 5.11 |
| >131.0 | 50 | 6.55 | (3.11-13.80) | 23.90 |

Univariate Cox PH analyses. Significance levels: 5% $\chi^2>3.84$; 1% $\chi^2>6.63$; 0.1% $\chi^2>10.83$.

Table V The prognostic value of each tumour marker (logarithmic form) when taking the Dukes stage into account

| | $\beta$ | s.e. ($\beta$) | $\chi^2$ Wald test |
|---|---|---|---|
| CEA | 0.165 | 0.069 | 5.66 |
| CA 19-9 | 0.272 | 0.089 | 9.22 |
| CA 50 | 0.272 | 0.062 | 19.43 |
| CA 242 | 0.217 | 0.055 | 15.40 |
| TPA | 0.553 | 0.110 | 25.52 |
| TPS | 0.550 | 0.105 | 27.22 |

Significance levels: 5% $\chi^2>3.84$; 1% $\chi^2>6.63$; 0.1% $\chi^2>10.83$.

a model that included CEA (data not shown). Adjustment for Dukes' stage reduced the significance of the variables. TPA was the most important variable after such an adjustment.

### Discussion

Every tested serum marker gave prognostic information in the group of patients of interest for pre- or peroperatively initiated additional treatment, i.e. patients in whom major surgery with curative intent was planned, revealing their possible importance for the selection of additional treatment. Also, in the group of patients of interest for prolonged post-operative adjuvant treatment, i.e. potentially cured patients (Dukes' stages A-C), the markers gave prognostic information. However, only some of them then kept their independent prognostic information, and without being highly significant.

We could confirm the potential value of the tumour markers CEA, CA 50 and TPA both for staging and for prediction of survival, which we previously reported in a separate study on rectal cancer only (Ståhle *et al.*, 1988*a*,*b*, 1989). We also found that the three other tumour markers, CA 19-9, CA 242 and TPS, can be used for the same purposes. However, the ability to predict either tumour stage or prognosis using these new markers did not exceed that of the older ones. The prognostic information of the best tumour marker, TPA, or of the best combinations of markers was not as high as that of the Dukes' stage. Further, when TPA was analysed in quartiles, only the highest one was significantly related to survival. Dukes' stage, in contrast, was of prognostic importance not only in the highest stage, even if, in this study, no statistically significant difference was disclosed between Dukes' stages A and B. Therefore, it might be said that the clinical impact of TPA, or any combination of tumour markers, is not similar to that of the Dukes stage. The information provided by the markers is, however, available before surgery.

The prognostic information provided by TPA alone and in relation to other serum markers and Dukes' stage has now been reported in two separate patient groups. The relation between the serum level of TPA and tumour stage was, however, not the same in this sample as in the previous study (Ståhle *et al.*, 1989). In the former study, which was somewhat larger and only composed of rectal cancer patients, the serum levels also discriminated between the early stages, and not only between Dukes' stages A-C and D. This discrep-

Table VI The relation between the preoperative serum levels (logarithmic form) and survival for the potentially cured patients ($n = 166$)

| Variable | Unadjusted for Dukes' stage | | | Adjusted for Dukes' stage | | |
|---|---|---|---|---|---|---|
| | $\beta$ | s.e. ($\beta$) | $\chi^2$ Wald test | $\beta$ | s.e. ($\beta$) | $\chi^2$ Wald test |
| CEA | 0.262 | 0.103 | 6.45 | 0.197 | 0.106 | 3.44 |
| CA 19-9 | 0.126 | 0.114 | 1.22 | 0.076 | 0.110 | 0.48 |
| CA 50 | 0.218 | 0.124 | 3.09 | 0.162 | 0.120 | 1.81 |
| CA 242 | 0.156 | 0.089 | 3.12 | 0.096 | 0.086 | 1.24 |
| TPA | 0.418 | 0.201 | 4.30 | 0.402 | 0.203 | 3.90 |
| TPS | 0.284 | 0.156 | 3.33 | 0.304 | 0.158 | 3.71 |

$\chi^2$ denotes a chi-square value (with one degree of freedom). Univariate Cox PH analyses. Significance levels: 5% $\chi^2 > 3.84$; 1% $\chi^2 > 6.63$; 0.1% $\chi^2 > 10.83$.

ancy is hard to explain, but might be due to the inclusion of patients with colon cancer. An increased preoperative serum level of TPA indicates generalised disease and suggests the need for a careful preoperative examination. Subclinical metastatic disease would be suspected, however even if other evidence of metastatic spread were negative, and additional treatment might be valuable. If this treatment were initiated post-operatively, the Dukes' stage could be used in the selection of patients. If, however, the additional treatment were initiated peroperatively (Taylor *et al.*, 1985; Metzger *et al.*, 1990), the selection could be aided by, for example, the preoperative serum level of TPA.

Preoperative irradiation of the pelvis is routinely given at our hospital to all patients with rectal cancer accepted for radical surgery (Påhlman and Glimelius, 1992) except when the tumour is revealed to be confined to the bowel wall by endosonography (i.e. Dukes' stage A) (Lindmark *et al.*, 1991). Irradiation is considered unnecessary in Dukes' stage A owing to the low risk of local recurrence and is of no real use in Dukes' stage D. The use of the preoperative value of TPA in the selection of rectal cancer patients for any preoperatively initiated treatment is not immediately apparent. A TPA level above the normal cut-off value indicates metastatic disease, and hence preoperative irradiation may appear superfluous. However, in that group, the 2-year survival exceeds 40%, which means that a number of patients are at risk of local recurrence with its known high morbidity. The majority of local failures occur within 2 years after surgery.

We conclude that new serum tumour markers should be investigated in multivariate systems to allow a proper evaluation of their possible clinical usefulness. Serum tumour markers may be of importance for the selection of patients for additional therapy in colorectal cancer, but the guidance they afford is far from absolute and their clinical usefulness not yet apparent.

**Acknowledgements**

This study was supported by grants from the Swedish Cancer Society (Project No. 1921-B92-10XAC) and the Swedish Medical Society.

**References**

CANGEMI V, VOLPINO P, FIORI E, GIAMMARCO A AND PIAT G. (1987). The role of tumour markers (CEA, TPA, CA 19-9) in colon and rectum carcinomas. *J. Nucl. Med. Allied Sci.*, **31**, 189-193.

CHU DZJ, ERICKSON CA, RUSSELL MP, THOMPSON C, LANG NP, BROADWATER RJ AND WESTBOOK KC. (1991). Prognostic significance of carcinoembryonic antigen in colorectal carcinoma. *Arch. Surg.*, **126**, 314-316.

COOPER EH, PURVES D, FINAN P AND PRIMROSE J. (1992). TPS in colorectal cancer. In *Tumor Associated Antigens, Oncogenes, Receptors, Cytokines in Tumor Diagnosis and Therapy at the Beginning of the Nineties*, Klapdor R. (ed.) pp. 28-29. Zuckschwerdt: Munich.

DUKES CE AND BUSSEY HJR. (1958). The spread of rectal cancer and its effect on prognosis. *Br. J. Cancer*, **12**, 309-320.

GUPTA MK, ARCIAGA R, BOCCI L, TUBBS R, BUKOWSKI R AND DEODHAR SD. (1985). Measurement of a monoclonal-antibody-defined antigen (CA 19-9) in the sera of patients with malignant and nonmalignant diseases. *Cancer*, **56**, 277-283.

HOLMGREN J, LINDHOLM L, PERSSON B, LAGERGÅRD T, NILSSON O, SVENNERHOLM L, RUDENSTAM C-M, UNSGAARD B, YNGVASON F, PETTERSSON S AND KILLANDER AF. (1984). Detection by monoclonal antibody of carbohydrate antigen CA 50 in serum of patients with carcinoma. *Br. Med. J.*, **288**, 1479-1482.

JASS JR, ATKIN WS, CUZICK J, BUSSEY HJR, MORSON BC, NORTHOVER JMA AND TODD IP. (1986). The grading of rectal cancer: historical perspectives and a multivariate analysis of 447 cases. *Histopathology*, **10**, 437-459.

JOHANSSON C, NILSSON O, BAECKSTRÖM D, JANSSON E-C AND LINDHOLM L. (1991). Novel epitopes of the CA 50-carrying antigen: chemical and immunohistochemical studies. *Tumor Biol.*, **12**, 159-170.

KOPROWSKI H, STEPLEWSKI Z, MITCHELL K, HERLYN M, HERLYN D AND FUHRER P. (1979). Colorectal carcinoma antigens detected by hybridoma antibodies. *Somatic Cell Genet.*, **5**, 957-972.

KUUSELA P, JALANKO H, ROBERTS P, SIPPONEN P, MECKLIN J-P, PITKÄNEN R AND MÄKELÄ, O. (1984). Comparison of CA 19-9 and carcinoembryonic antigen (CEA) levels in the serum of patients with colorectal diseases. *Br. J. Cancer*, **49**, 135-139.

LAWLESS JF. (1982). Statistical Models and Methods for Life-time Data. Wiley: New York.

LEWI H, BLUMGART LH, CARTER DC, GILLIS CR, HOLE D, RATCLIFFE JG, WOOD CB, MCARDLE CS. (1984). Pre-operative carcinoma-embryonic antigen and survival in patients with colorectal cancer. *Br. J. Surg.*, **71**, 206-208.

LINDMARK G, ELVIN A, PÅHLMAN L AND GLIMELIUS B. (1992). The value of endosonography in preoperative staging of rectal cancer. *Int. J. Colorect. Dis.*, **7**, 162-166.

METZGER U, LAFFER U, AEBERHARD P, ARIGONI M, ARMA S, BARRAS J, EGELI R, MARTINOLI S, MUELLER W AND SCHWEIZER W. (1990). Randomized multicenter trial of adjuvant intraportal chemotherapy for colorectal cancer. *Acta Chir. Scand.*, **156**, 467-474.

MOERTEL CG, O'FALLON JR, GO VLW, O'CONNELL MJ AND THYNNE GS. (1986). The preoperative carcinoembryonic antigen test in the diagnosis, staging, and prognosis of colorectal cancer. *Cancer*, **58**, 603-610.

MOERTEL CG, FLEMING TR, MACDONALD JS, HALLER DG, LAURIE JA, GOODMAN PJ, UNGERLEIDER JS, EMERSON WA, TORMEY DC, GLICK JH, VEEDER MH AND MAILLIARD JA. (1990). Levamisole and fluorouracil for adjuvant therapy of resected colon carcinoma. *N. Engl. J. Med.*, **322**, 352-358.

MOORE M, JONES DJ, SCHOFIELD PF AND HARNDEN DG. (1989). Current status of tumor markers in large bowel cancer. *World J. Surg.*, **13**, 52-59.

NILSSON O, JOHANSSON C, GLIMELIUS B, PERSSON B, NÖRGAARD-PETERSEN B, ANDRÉN-SANDBERG Å AND LINDHOLM L. (1992). Sensitivity and specificity of CA 242 in gastrointestinal cancer. A comparison with CEA, CA 50 and CA 19-9. *Br. J. Cancer*, **65**, 215-221.

PUTZKI H, STUDENT A, JABLONSKI M AND HEYMANN H. (1987). Comparison of the tumor markers CEA, TPA, and CA 19-9 in colorectal carcinoma. *Cancer*, **59**, 223–226.

PÅHLMAN L AND GLIMELIUS B. (1992). Preoperative and postoperative radiotherapy and rectal cancer. *World J. Surg.*, **16**, 858–863.

STÅHLE E, GLIMELIUS B, BERGSTRÖM R AND PÅHLMAN L. (1988*a*). Preoperative serum markers in carcinoma of the rectum and rectosigmoid. II. Prediction of prognosis. *Eur. J. Surg. Oncol.*, **14**, 287–296.

STÅHLE E, GLIMELIUS B, BERGSTRÖM R AND PÅHLMAN L. (1988*b*). Preoperative serum markers in carcinoma of the rectum and rectosigmoid. I. Prediction of tumour stage. *Eur. J. Surg. Oncol.*, **14**, 277–286.

STÅHLE E, GLIMELIUS B, BERGSTRÖM R AND PÅHLMAN L. (1989). Preoperative prediction of outcome in patients with rectal and rectosigmoid cancer. *Cancer*, **63**, 1831–1837.

TAYLOR I, MACHIN D, MULLEE M, TROTTER G, COOKE T AND WEST C. (1985). A randomized controlled trial of adjuvant portal vein cytotoxic perfusion in colorectal cancer. *Br. J. surg.*, **72**, 359–363.